# 

**Published:** 1889-04-27

**Authors:** 


SATURDAY, APRIL 27th, 1889.

**Prince, Princess, and People**—The Prince - - - 49
" " " The Princess - - - 50
" " " The People - - - 51
**Poem—The Royal Hand** - - - 52
**The Public Life of the Prince and Princess of Wales** - - - 53
Politics or Philanthropy - - - 53
The Prince Consort's Legacy - - - 54
Love for Children - - - 54
The Working Men's Exhibition - - - 55
Charities of the Three Kingdoms - - - 56
The Value of Festival Dinners - - - 57
Death of the Princess Alice - - - 58
Hospitals for Consumption - - - 59
Women's Hospitals - - - 59
The International Medical Congress - - - 60
The Abolition of Slavery - - - 61
The Torbay Fishermen - - - 61

**EXTRA SUPPLEMENT—THE NURSING MIRROR.**

**En Passant.**—Reading for Nurses—Hypnotism—Registration of Midwives—The Clothing of New-born Children—Hymn by Canon Kingsley—A Defence of Asylum Nurses - i
**Sick Children in their Homes** - - - ii
**Patients, not Prisoners** - - - ii
**Appointments and Vacancies** - - - ii

